# VagiBIOM *Lactobacillus* suppository improves vaginal health index in perimenopausal women with bacterial vaginosis: a randomized control trial

**DOI:** 10.1038/s41598-024-53770-1

**Published:** 2024-02-09

**Authors:** Vijitha Vivekanandan, Zaiba Hasan Khan, Giriprasad Venugopal, Bhavana Musunuru, Priyanka Mishra, Shalini Srivastava, Balamurugan Ramadass, Bobban Subhadra

**Affiliations:** 1 Biom Pharmaceutical Corporation, 2203 Industrial Blvd, Sarasota, FL USA; 2 Center of Excellence for Clinical Microbiome Research (CCMR), All India Institute of Medical Sciences, Bhubaneswar, Odisha, India; 3 Vedic LifeSciences, Clinical Research, Andheri (West), Mumbai, Maharashtra India; 4 Department of Biochemistry, All India Institute of Medical Sciences, Bhubaneswar, Odisha, India

**Keywords:** VagiBIOM, *Lactobacillus* suppository, Vaginal health index, Bacterial vaginosis, Perimenopause, Microbiology, Biomarkers, Diseases, Medical research, Pathogenesis

## Abstract

Bacterial vaginosis (BV) can cause vaginal dysbiosis that may influence general vaginal health and pregnancy complications. Balancing vaginal microbiome using *Lactobacillus* spp. may be a new way to prevent and treat mild BV. We conducted a randomized, double-blind, placebo-controlled pilot study aimed at evaluating the effect of the product VagiBIOM, a multi-*Lactobacillus* vaginal suppository, on peri- and premenopausal women with BV in restoring vaginal pH and overall vaginal health by resetting the vaginal microbiome composition. Sixty-six peri- and premenopausal women with BV symptoms were randomized with a 2:1 ratio to be treated with VagiBIOM or placebo suppositories. Vaginal pH, VAS itching score, total Nugent score, and vaginal health index (VHI) were measured. Vaginal microbiome changes before and after the treatment were analyzed by 16S rRNA sequencing and bioinformatics analysis. After 4 weeks of intervention with VagiBIOM or a placebo, the mean score for vaginal pH, VAS itching, and total Nugent score was significantly decreased from the baseline. Compared to the baseline scores, the VHI scores improved significantly following 28-day intervention (*p* < 0.001). Our results revealed two *Lactobacillus* species, *L. hamsteri,* and *L. helveticus*, as indicator species occurring differentially in the VagiBIOM-treated group. Furthermore, the regression and species network analyses revealed significant bacterial associations after VagiBIOM treatment. *Lactobacillus hamsteri* was positively associated with the Nugent score and negatively associated with vaginal pH. *L. iners* and *L. salivarius* were positively and inversely associated with VHI. As is typical, *Bacteroides fragilis* was positively associated with vaginal pH and negatively associated with the Nugent score. Interestingly, the *Lactobacillus* spp. diversity improved after VagiBIOM treatment. The VagiBIOM suppository treatment for peri- and premenopausal women with BV significantly relieved vaginal itching by decreasing vaginal pH and Nugent scores and improving the overall VHI after 4 weeks’ intervention. This effect was primarily the result of VagiBIOM improving vaginal *Lactobacillus* diversity.

*Trial Registration* ClinicalTrials.gov registration: NCT05060029, first registration 09/28/2021: Title: A Pilot Study to Evaluate the Efficacy and Safety of Lactobacillus Species Suppositories on Vaginal Health and pH.

## Introduction

Vaginitis is a disorder that causes the vagina to become infected or inflamed and is often caused by infectious organisms such as pathogenic bacteria, yeast or viruses or irritation from chemicals^1,2^. Atrophic vaginitis frequently affects people transitioning to menopause, where the lining of the vagina becomes drier and thinner from a lack of estrogen^3^. Infectious vaginitis is often treated with antimicrobials; however, because of the dynamic nature of vaginal tissues (e.g., menstrual cycle, pregnancy, etc.), effective treatment of vaginal infections with conventional antibiotics and antifungals often comes with challenges such as unavoidable dose-related side effects with an increased risk of bacterial resistance. Because of this, most vaginal infections are recurring in nature^4,5^.

The vaginal microbiome is the first line of defense against vaginal infection due to the competitive exclusion of pathogenic microbes^6^. The vagina remains relatively stable until a woman reaches puberty, after which the hormonal changes cause the colonization of *Lactobacillus* in the vaginal environment^7^. The reduction of circulating female hormone levels in women approaching menopause triggers various physiological changes in the vagina. Vulvovaginal atrophy, dryness, itchiness, redness, loss of elasticity, inflammation, and atypical secretions are changes in the vagina as estrogen and progesterone levels decrease in the circulation^8^. *Lactobacillus crispatus* is among the most prevalent species of the *Lactobacillus*-dominated human vaginal microbiota^9^ that develops with vaginal tissues to restrict the growth of other bacteria’s associated with vaginal dysbiosis, thereby reducing the susceptibility to adverse sequelae^10^. Any alterations in the vaginal microbiota can cause symptomatic conditions. These conditions may include Bacterial vaginosis (BV), vaginal candidiasis, and trichomoniasis^11^. BV is a common outcome, often asymptomatic dysbiosis of the human vagina characterized by an imbalance in the normal vaginal microbiota due to loss of lactobacilli and an overgrowth of certain anaerobic bacteria^12^.

Perimenopausal women have a high chance of accumulating BV-associated microbes. The reduction in estrogen leads to a decline in glycogen levels, reducing the *Lactobacillus* concentration in the vaginal environment during menopause^13^. *Lactobacillus* spp. produce lactic acid, which creates an acidic vaginal environment conducive to *Lactobacillus* growth while preventing the propagation of other anaerobic bacterial species.

Moreover, *Lactobacillus* also inhibits pro-inflammatory cytokine induction by promoting homeostasis^14–16^. Probiotic activity results from the action of individual *Lactobacillus* spp. and by a consorted effect of multispecies interactions or the formation of a dominant species network^17^. The overabundance of *Gardnerella vaginalis* and other anaerobes usually causes BV. Gram stain is a widely used method for diagnosing BV and has been used since 1965^18^. When used for BV diagnosis, Gram stain results include *Gardnerella* morphotypes, such as cocci, fusiform, curved rods, and reduced numbers of *Lactobacillus* morphotypes^19^. The Nugent score based on Gram stain serves as a valid criterion for identifying cases of BV^20^.

Most pharmacological management of BV includes oral and topical antibiotics such as metronidazole, clindamycin, and fluconazole. Antibiotics inhibit the growth of BV-associated microbes that support *G. vaginalis* and other microbes without affecting *Lactobacillus*. However, frequent antibiotic use may lead to antibiotic resistance and undesirable side effects such as thrush, dizziness, rash, and nausea^21^. Moreover, the cure rates are reduced with multiple antibiotic use, and the BV recurrence rates have been shown to reach as high as 80%^22^. Recently, probiotic microbiome therapy has been explored as an adjunct or replacement therapy for antibiotic treatment to prevent BV and other infections. These probiotics usually contain beneficial *Lactobacillus* and help maintain a healthy vaginal microbial environment. Most of the probiotics on the market are consumed orally^23^. Vaginal suppositories are a relatively new topical administration of active compounds that are used to treat urogenital vaginal infections and other local diseases due to the ease and targeted administration^24–26^. A few *Lactobacillus*-containing suppositories have been explored to treat and prevent BV^27,28^.

So far, studies have failed to measure the spatial and temporal vaginal microbiome changes in BV post-intervention. We hypothesized that the *Lactobacillus* in vaginal suppositories lowers the vaginal pH by producing lactic acid to the ideal pH range of 3.5–4.5 and destabilize the colonization or establishment of BV-associated microbes network by resetting the healthy vaginal microbiome^29^. Our study has used the ‘VagiBIOM’ product, which primarily based on the LiveBiom™ fermentation technology, a patented process to create commensal microbiome biofilms in a predigested prebiotic fiber matrix. The technology uses targeted evolution of strains through interaction with host tissues to yield a human-tissue-adapted probiotic–prebiotic complex^30^, which address the temporal vaginal health index and microbiome changes in perimenopausal women. Therefore, our study aimed to evaluate the effect of VagiBIOM, an off-the-shelf novel combination of *L. crispatus* Bi16*, L. gasseri* Bi19*, B. coagulans* Bi34*, L. acidophilus* Bi14, and a prebiotic-based vaginal suppository in peri- and premenopausal women, on vaginal health and microbiome changes.

## Methods

### Study design and execution plan

This was a prospective, randomized, double-blind, placebo-controlled pilot study to evaluate VagiBIOM: a *Lactobacillus* suppository for improving the vaginal health index in perimenopausal women with BV who were visiting the hospitals at Jaipur and Varanasi, India. Vedic Lifesciences, Mumbai, India, conducted the clinical trial (ClinicalTrials.gov registration: NCT05060029, first registration 28/09/2021), and the bacterial samples were sequenced at the Clevergene, Bangalore (Project ID: CG_DN_1816) and data was analyzed at the Center of Excellence for Clinical Microbiome Research at AIIMS Bhubaneswar. Vedic Lifesciences, Pvt Ltd. obtained ethics committee approval from Harmony Ethical Research Committee, Shree Hospital, Mumbai-400071, Maharashtra, India (Reg. No. ECR/1411/Inst/MH/2020), Institutional Ethics Committee, JNU Institute for Medical Sciences and Research Centre, Jaipur-302017, Rajasthan, India (Reg. No. ECR/905/Inst/RJ/2017/RR-20) and Shubham Sudbhawana Superspeciality Hospital Ethics Committee, Shubham Sudbhawana Superspeciality Hospital, Varanasi-221005, Uttar Pradesh, India (Reg. No. ECR/667/Inst/UP/2014/RR-20) with a Study ID BP/210503/VB/VDPH. All the procedures and protocols used in this research was in accordance with the Declaration of Helsinki.

### Participant characteristics

The sample size for the present study was calculated for the superiority trial using https://riskcalc.org/samplesize/. Ninety-two female participants aged ≥ 40 and ≤ 65 years were screened. A patient information sheet in their native language was provided, and the study protocol and primary/secondary outcome measures were explained orally and written informed consent was obtained from all subjects and/or their legal guardian(s). Non-pregnant, non-breastfeeding females between the age of 40–65 years, with homogeneous, thin, white discharge that smoothly coats the vaginal walls, presence of the clue cells, fishy odor of vaginal discharge, a Nugent score of ≥ 7, vaginal fluid pH ≥ 5, with a total Vaginal Health Index (VHI) score of < 15 were included in the study. Those participants undergoing hormone replacement therapy (HRT) or taking prebiotics/probiotics or any antibiotics 1 month before screening were excluded from the study (detailed description in Supplementary File 1). Based on inclusion and exclusion criteria, 46 participants were assigned to the VagiBIOM intervention group, and 20 were assigned to the placebo group (coconut oil fatty acid). Vaginal pH, visual analog scale (VAS) itching score, total Nugent score, and vaginal health index (VHI) were assessed for each participant, with follow-up assessments on day 7, day 21, and day 28.

### Study interventional product

VagiBIOM is a registered formulation that contains different *Lactobacillus* spp. and comprises 1–2% *L. crispatus* Bi16*, L. gasseri* Bi19*, B. coagulans* Bi34*, L. acidophilus* Bi14, coconut oil fatty acids, prebiotic complex (0.05–2%), hyaluronic acid (0.1–0.3%), silica gel (0.1–0.3%), and lactic acid (0.01–0.025). We have packaged these probiotic–prebiotic complex (0.25 g) in a slow-release suppository fatty acid base with a melting point of 36–38 °C. The preformed solid dosage formulation was then enclosed in a rapid-release fatty acid suppository (2.0 g) with a melting point of 32–34 °C. The unique mixtures of mono-, di-, and tri-acyl glycerides came from coconut oil (99% triglycerides with free fatty acids (less than 0.2%) and lauric, caprylic, and capric acids). Coconut oil fatty acid (100%) was used as the placebo for the study. All participants were instructed to insert one suppository daily (0.25 g) before bedtime for 28 days.

### Randomization and method of assigning participants to study groups

First, participant IDs were generated and printed on the product labels with other appropriate information. A statistician who was not directly involved in this study prepared the randomization chart. StatsDirect Software version 3.1.17^31^ was utilized for block randomization. The participants were randomized in blocks of 6 in a 2:1 ratio to receive either VagiBIOM or a placebo. The kits were dispensed as per the randomization chart, and this chart was maintained in the designated study folder with limited access.

### Vaginal swabs, DNA isolation, and microbiome analysis

Vaginal swabs were collected at baseline (Day 0) and after intervention (Day 28) from the VagiBIOM group (n = 14) and placebo group (n = 8) using sterilized cotton swabs from the vaginal pH kit and were stored at − 80 °C. DNA was isolated from the 44 samples using a Powerlyser Powersoil kit (QIAGEN, Germany) as a modified protocol and was quantitated using a NanoDrop spectrophotometer (NanoDrop™ 2000, Thermo Scientific™, USA). Briefly, the vaginal swab sample was transferred to a bead vial, followed by the addition of 750 µL of bead solution and 60 µL of solution C1. The vial was vortexed and subjected to 65 °C for 15 min in a water bath, followed by bead beating for 2 successive cycles. The remaining steps were followed according to the manufacturer's guidelines. The vaginal microbiome was assessed by 16S rRNA gene sequencing (V3–V4 region) using a previously published protocol from our group^32^, and the Illumina MiSeq platform was used for 16S rRNA amplification.

### Methods of vaginal examinations:

#### Vaginal pH

The participants were asked to change into a medical gown. Sterilized cotton swabs from the vaginal pH kit were used for vaginal swab sample collection. After 10–20 s, a color change in the cotton swab was detected, the pH values were compared with the vaginal pH test card, and the pH of the vaginal environment was recorded.

#### VAS itching score

The 0–10 VAS scale for the itching diary was dispensed to the participants during the study visit. The participants were instructed to circle a score from 0 to 10, corresponding to the vaginal itch severity they had experienced over the past week. The itch severity was assessed using this scale, and the efficacy of the investigational product compared to that of the placebo was determined.

#### Total Nugent score

A sterile cotton swab was inserted into the vaginal opening, and the swab was rotated inside for 10–30 s. The swab was removed without touching the skin and stored inside the tube used for swab collection. The swab sample was stored at 2–8 °C until it reached the processing center. The sample reached the processing center within 48 h of sample collection and was processed within 6 h of receiving the swab sample.

An overall vaginal assessment was also performed, and each criterion, including vaginal wall elasticity, secretion, pH, epithelial mucosa, and moisture, was scored from 1 to 5 based on the clinical examination. The responder's analysis using clinical cure rate (CCR), the ratio of patients who have achieved a clinical cure to the total number of patients in the study, was calculated and expressed as a percentage.

### Statistical and bioinformatics analysis

Clinical parameters, such as vaginal pH, VHI, VAS itching score, and total Nugent score were assessed at various time points: baseline, Day 7, Day 14, and Day 28 and compared for the VagiBIOM and placebo groups. Using the R package, a *t*-test was applied for statistical comparison between the VagiBIOM and placebo groups, and *p* < 0.05 was considered significant. The bioinformatics analysis trimmed the reads (20 bp) from the 5′ end to remove the degenerate primers. The trimmed reads were processed to remove adapter sequences and low-quality bases using Trimgalore (https://www.bioinformatics.babraham.ac.uk/projects/fastqc/). The quality control (QC) passed reads were imported into MOTHUR^33^, and the pairs were aligned to form contigs.

The contigs were screened for errors, and only those between 300 and 532 bp were retained. Any contig with ambiguous base calls was rejected. The high-quality contigs were checked for identical sequences, and duplicates were merged. Although the primers for the experiment were designed for 16S bacterial rRNA, there is a good chance that nonspecific amplification of other regions will occur. To correct for this, we aligned the contigs to the GREENGENES V.13.9–99 database for 16S rRNA^34^. Depending on the variable region being amplified, most contigs will align to their respective region on the database. Any ambiguous contigs aligning with other regions in the database were discarded. After this process, the gaps and the overhang at the ends of the contigs and chimera formed due to PCR errors were removed. The UCHIME algorithm^35^ was used to flag contigs with chimeric regions. A known reference of all the chimeric sequences was used to identify and remove possible chimeric sequences. The filtered contigs were processed and classified into taxonomic outlines based on the GREENGENES. The contigs were clustered into operational taxonomic units (OTUs) with a 97% cutoff value. After the classification, OTU abundance was estimated for each of the samples. The alpha diversity at the species level between the study groups was determined using the observed richness, Shannon index, Simpson index, and Pielou's evenness alpha diversity measures. The fold difference between the baseline and post-intervention samples was calculated for all alpha indices by converting natural values into log2. The outcomes of the VagiBIOM and placebo groups were compared using a *t*-test. All statistical analyses were conducted in R^36^.

### Indicator species analysis

We performed indicator species analysis in R using an indicspecies package based on the function multipatt to identify microbial species found more often in one group than another^37^. This function is a multilevel pattern analysis that calculates an indicator value for each species in association with the input groups and then finds the group with the highest association with each species. We analyzed VagiBIOM and the placebo between the baseline and 28 days (post-treatment). Finally, the statistical significance of this relationship was tested using a permutation test.

### Regression analysis

We conducted regression analysis using efficacy parameters and the top 20 highly abundant microbial species representing approximately 97% of the species data to determine the relationship between microbes and efficacy parameters (vaginal pH, VHI, total Nugent score, VAS itching). We also performed regression on *Lactobacillus* spp. present in the data with efficacy parameters to determine the relationship between *Lactobacillus* and these parameters.

### Species network construction

We applied Spearman correlation to analyze the associations of the microbial species with the efficacy parameters for the top 20 most abundant species in the VagiBIOM and the placebo groups using the R corr package. For this, microbial species counts were taken, and a centered log ratio (CLR) was applied for transformation because microbiome data are compositional and zero-inflated. The correlation was used to construct a species network using the Cytoscape tool^38^.

### Ethics approval and consent to participate

Vedic Lifesciences, Pvt Ltd. obtained Ethics committee approval from Harmony Ethical Research Committee, Shree Hospital, Mumbai-400071, Maharashtra, India (Reg. No. ECR/1411/Inst/MH/2020), Institutional Ethics Committee, JNU Institute for Medical Sciences and Research Centre, Jaipur-302017, Rajasthan, India (Reg. No. ECR/905/Inst/RJ/2017/RR-20) and Shubham Sudbhawana Superspeciality Hospital Ethics Committee, Shubham Sudbhawana Superspeciality Hospital, Varanasi-221005, Uttar Pradesh, India (Reg. No. ECR/667/Inst/UP/2014/RR-20) with a Study ID BP/210503/VB/VDPH.

## Results

**Figure 1:**
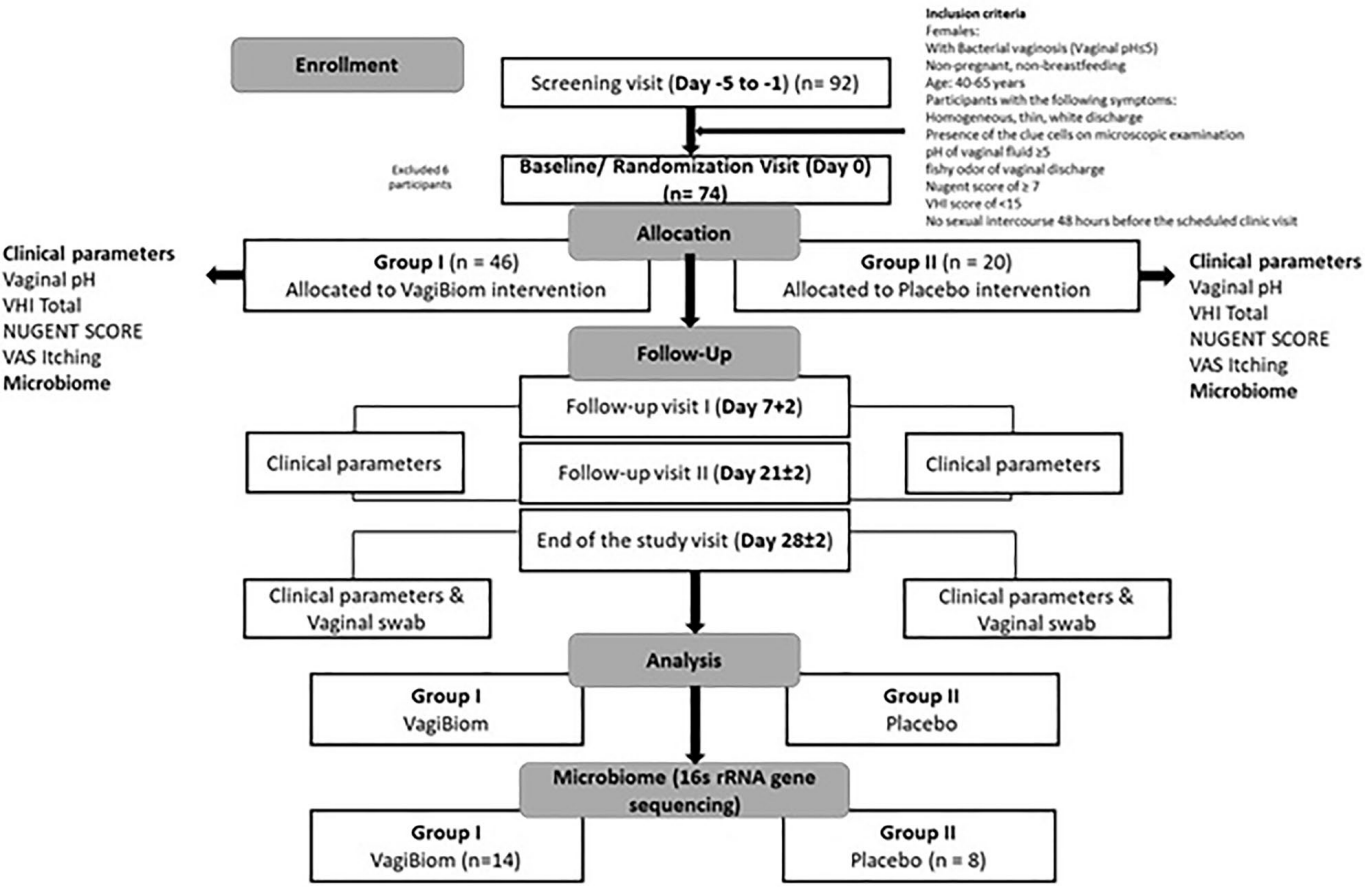
Consort flow diagram for the screening and enrollment of study participants.

To determine how VagiBIOM *Lactobacillus* suppository support vaginal health, we analyzed VHI, vaginal pH, VAS itching, Nugent scores, and vaginal microbiome changes in a randomized, double-blind, placebo-controlled clinical evaluate the effect of the product VagiBIOM on BV. The consort diagram for the study is illustrated in Fig. 1. Of the 92 patients screened, 66 participants with a mean age of 46 ± 5.57 (mean ± SD) years were recruited for the study. Forty-six and twenty patients were allocated to the VagiBIOM and placebo treatment groups, respectively. The patient demographics are presented in Table 1.

**Table 1:** Demographic and clinical parameters of the study participants in the VagiBIOM (treatment) and placebo (control) groups.

Characteristics	VagiBIOM (n = 46)	Placebo (n = 20)
Age (Years ± SD)	46 ± 5.29	47 ± 6.27
Clinical parameters		
Bacterial Vaginosis (BV) diagnostic score	4 ± 0.48	4 ± 0.47
Vaginal pH	6 ± 0.62	6 ± 0.75
Total vaginal health index (VHI)	12 ± 1.47	12 ± 1.5
Total nugent score	8 ± 0.76	8 ± 0.71
Visual analog scale (VAS) Itching	6 ± 2.7	7 ± 2.96
Demography		
Body Mass Index (BMI) (kg/m^2^ ± SD)	24 ± 3.56	25 ± 2.92

The values are presented as the mean ± standard deviation.

### VagiBIOM mediated significant changes in VHI, vaginal pH, VAS itching, and Nugent scores

**Figure 2:**
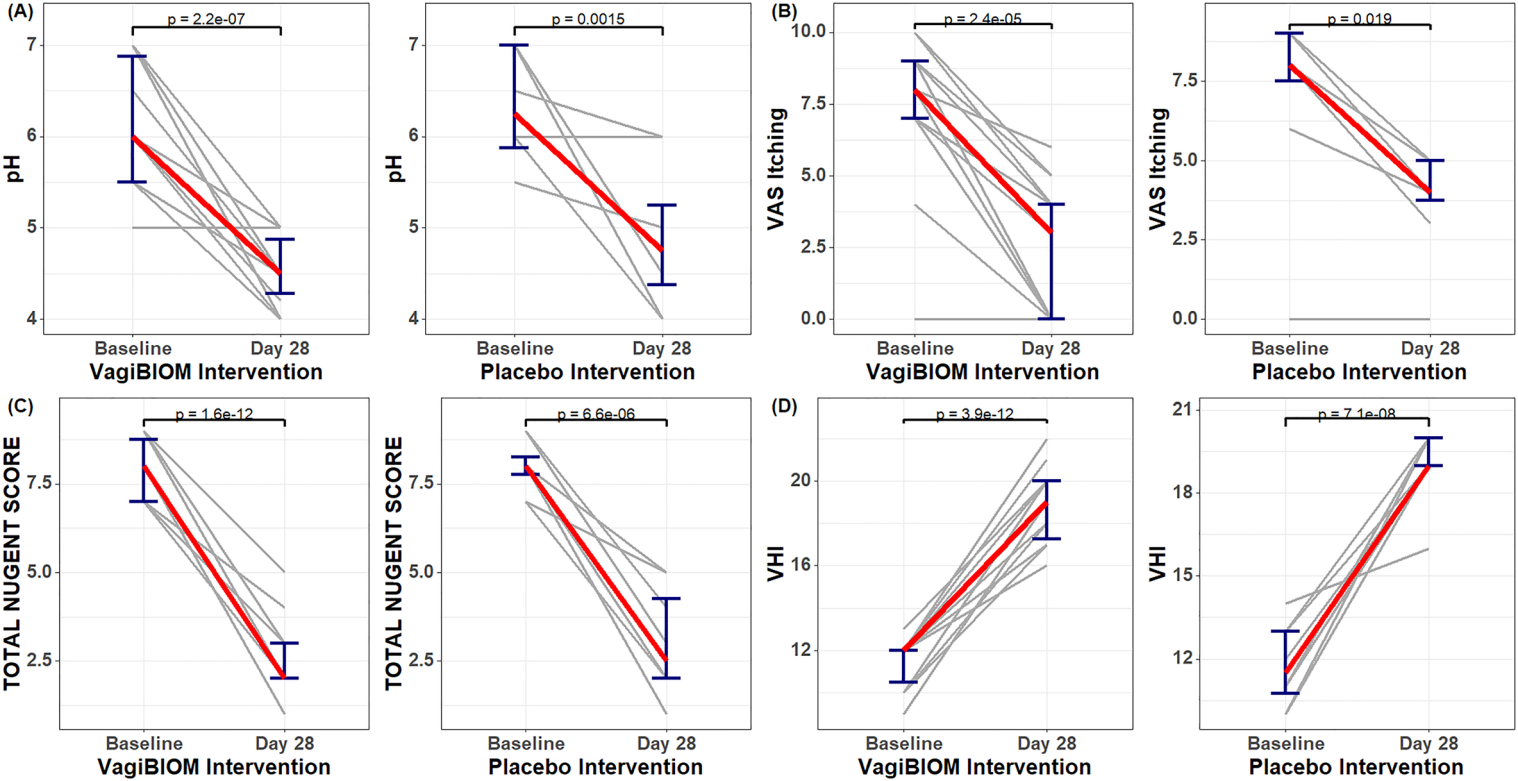
Comparison of efficacy parameters between baseline and post-intervention (Day 28) for the VagiBIOM and placebo groups. (**A**) Vaginal pH comparison (**B**) VAS itching (**C**) Total Nugent score (**D**) VHI. *VAS, Visual Analog Scale; VHI, Vaginal Health Index.

In the VagiBIOM treatment group, there was a substantial decrease in the pH from Day 7 to Day 21 and further from Day 21 to Day 28 as the vaginal pH value dropped significantly from 5.5 to 5.0 and then 5.0 to 4.5, *p* =  < 0.001, respectively (Fig. 2A, Tables 2 and S1). The VAS itching score, the total Nugent score, and the VHI score dropped considerably among the four different time points (Fig. 2B–D). Similar results were also observed in the placebo group (Table 2, Supplementary Tables S2–S4). The CCR in the VagiBIOM group compared to the placebo group was 76.08% vs. 40% (the Nugent score cutoff < 4, *p* < 0.006) and 82.6% vs. 45% (vaginal pH cutoff ≤ 4.5, *p* < 0.05). Therefore, these results reinforces our hypothesis that VagiBIOM show beneficial effect on the measured parameters.

**Table 2:** Significant changes in clinical parameters from baseline to post-intervention (Day 28).

Clinical parameters	Placebo (n = 20)		*p* Value (< 0.05 taken as a significant)	VagiBIOM (n = 46)		*p* Value (< 0.05 taken as a significant)
	Pre	Post		Pre	Post	
Vaginal pH	6.0 ± 0.75	4.9 ± 0.55	< 0.001	5.9 ± 0.68	4.5 ± 0.40	< 0.001
Total Vaginal health Index (VHI)	12 ± 1.5	19 ± 1.74	< 0.001	12 ± 1.47	19 ± 2.25	< 0.001
Total Nugent score	8 ± 0.71	4 ± 1.46	< 0.001	8 ± 0.77	3 ± 1.22	< 0.001
Visual analog scale (VAS) itching	7 ± 2.96	3 ± 1.93	< 0.001	6 ± 2.7	3 ± 1.81	< 0.001

The values are presented as the mean ± standard deviation.

### Alteration of vaginal microbiome composition and diversity

**Figure 3:**
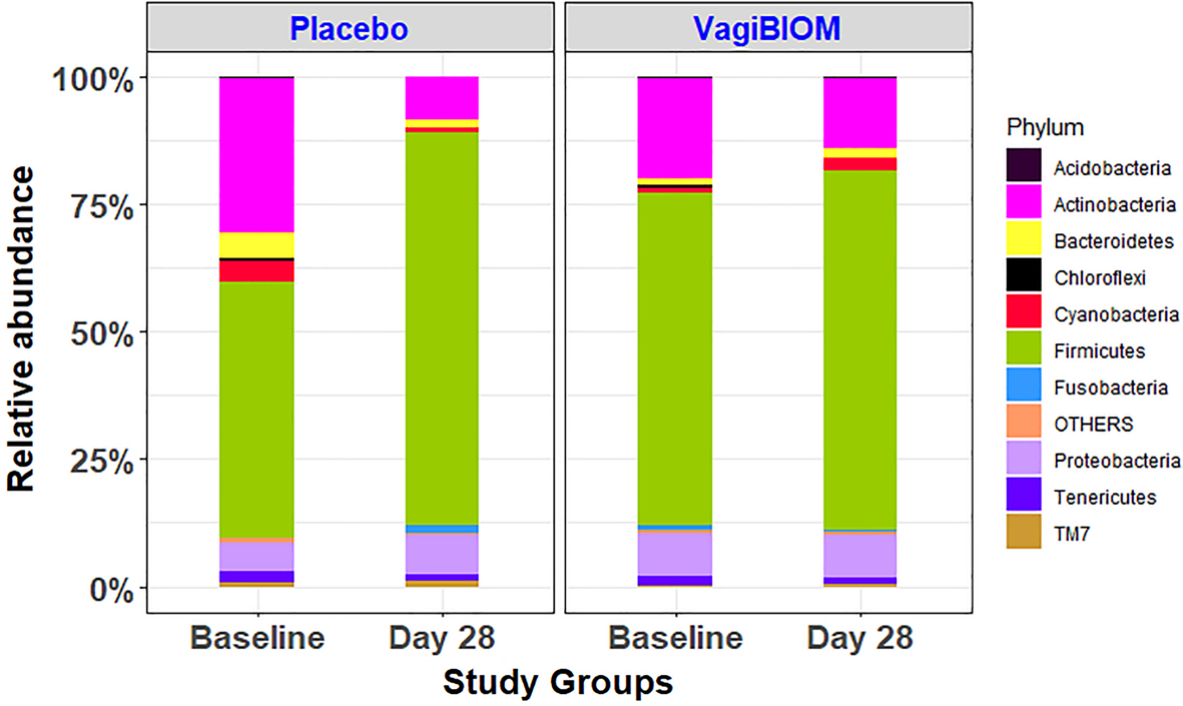
Microbiome composition in BV: relative abundance of the top 10 bacterial taxa at the species level in the placebo vs. VagiBIOM groups at baseline and post-intervention (Day 28).

**Figure 4:**
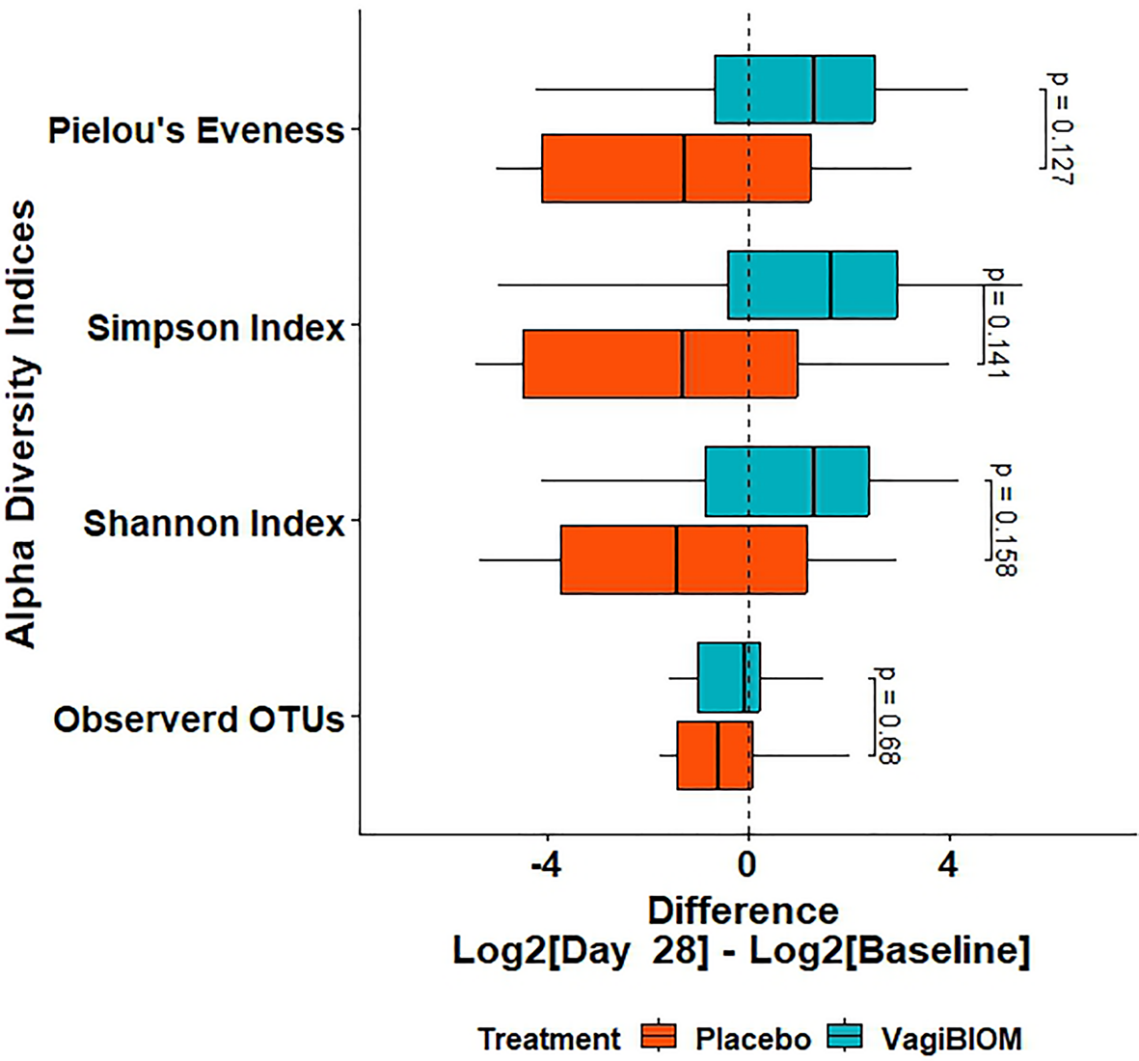
Differential abundance of species richness and diversity in placebo vs. VagiBIOM-treated groups.

The vaginal microbiome composition was analyzed by sequencing the V3–V4 hypervariable regions from 44 vaginal swabs including pre and post-treatment: VagiBIOM group (n = 14) and placebo group (n = 8). After quality control and step-wise filtering process, 89,17,810 high-quality sequences with an average length of 301 bp were recovered for further analysis, with an average of ~ 1,93,865 reads per sample (ranging from 17,770 to 5,91,506). Among the top 10 phyla, Firmicutes and Proteobacteria were the most abundant and Actinobacteria was found to be less abundant post-treatment in VagiBIOM and Placebo (Fig. 3). Compared to the placebo treatment, the VagiBIOM treatment increased fold differences, however was not statistically different (Fig. 4).

### Indicator species in the VagiBIOM and placebo groups

**Figure 5:**
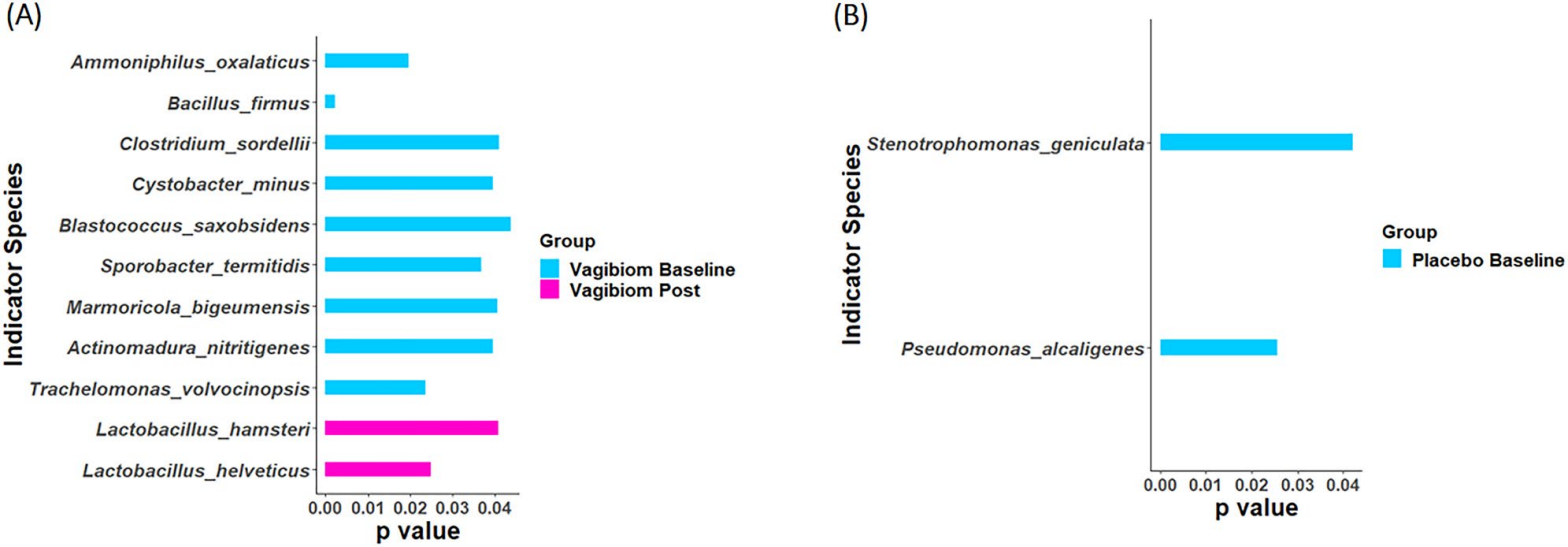
Indicator species (**A**) VagiBIOM group (**B**) Placebo group.

The taxa present in at least 20% of the study samples were considered for indicator species analysis. Based on this criterion, 175 species out of 647 were selected and tested for indicator species analysis. In the post-VagiBIOM treatment group (Fig. 5A), only two *Lactobacillus* spp.*, L. hamsteri,* and *L. helveticus* were significant indicators, while no indicators were found in the post-placebo treatment group (Fig. 5B). At the VagiBIOM baseline, multiple microbes atypical of the niche were identified; they were *Trachelomonas volvocinopsis*, *Actinomadura nitrigenes*, *Marmoricola bigeumensis*, *Sporobacter termiditis*, *Blastococcus saxobsidens*, *Cystobacter minus*, *Clostridium sordellii*, *Bacillus firmus*, and *Ammonophilus oxalaticus*, and at the placebo baseline, only *Pseudomonas alcaligenes* and *Stenotrophomonas geniculata* were found as indicator species.

### Regression analysis

**Figure 6:**
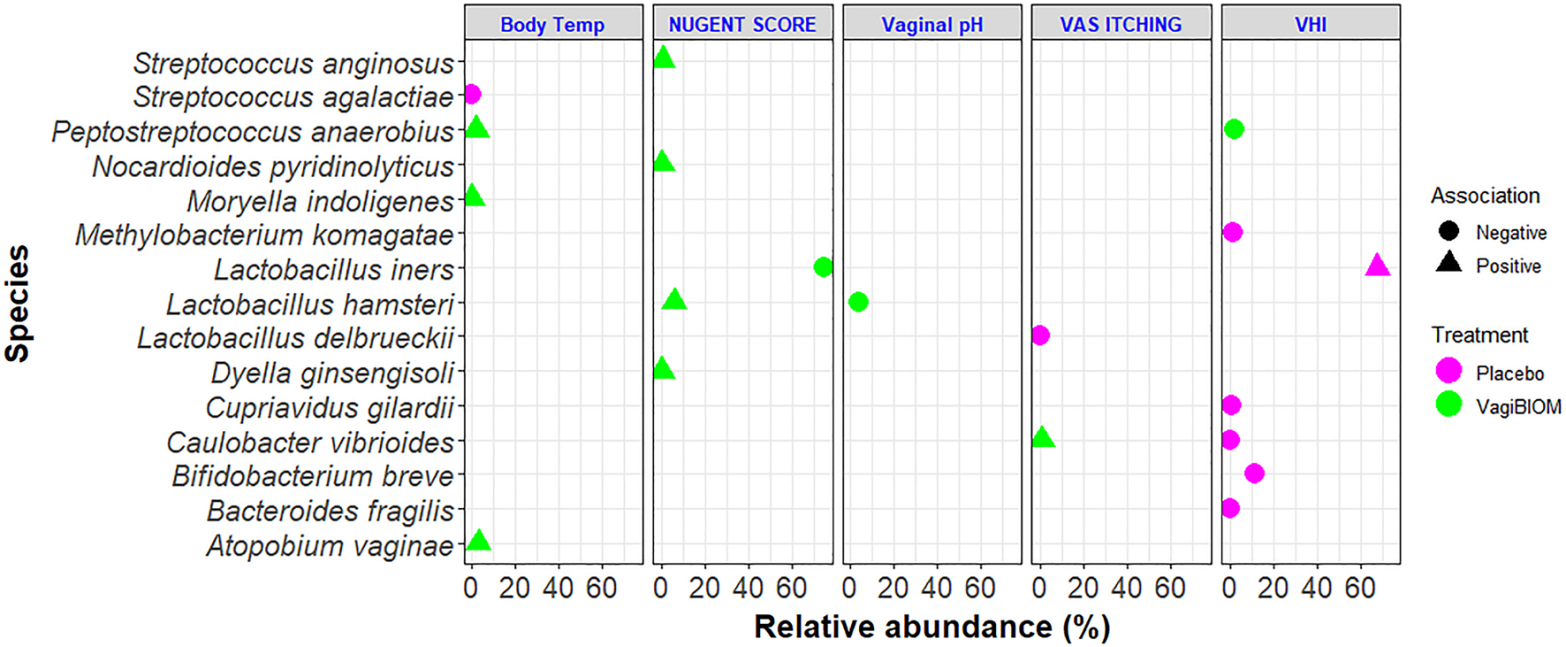
Regression analysis between the top 20 species and efficacy parameters in the VagiBIOM and placebo-treated groups. *Bubble points denote the relative abundance of each species; color denotes the type of treatment; triangle denotes positive association; circle denotes negative association. *Abbreviations* Body Temp, Body temperature; VAS, Visual analogue scale; VHI, Vaginal health index.

The results of the regression analysis for the top 20 species and clinical parameters showing only a significant association with the post-treatment group are presented in Fig. 6. In the VagiBIOM post-treatment, *Moryella indoligenes*, *Peptostreptococcus anaerobius,* and *Atopobium vaginae* had a positive association with body temperature; *Caulobacter vibrioides* showed a positive association with VAS itching; *Dyella ginsengisoli*, *L. hamsteri*, *Nocardioides pyrimidinolyticus*, and *Streptococcus anginosus* were found to have a positive association with the Nugent score (1 to 5), while only *Peptostreptococcus anaerobius* showed a negative association with VHI. On the other hand, *L. hamsteri,* the indicator species, was negatively associated with vaginal pH, and *L. iners* was negatively associated with the Nugent score. Likewise, in the placebo-treated group, a positive association was found between *S. agalactiae* and body temperature, and *Bacteroides fragilis*, *Bifidobacterium breve*, *Cupriavidus gilardii*, *Caulobacter vibrioides*, and *Methylobacterium komagatae* were negatively associated with VHI. On the other hand, *L. delbreuckii* had a negative association with VAS itching, and *L. iners* had a positive association, indicating the potential role these microbes had in the progression of BV.

**Figure 7:**
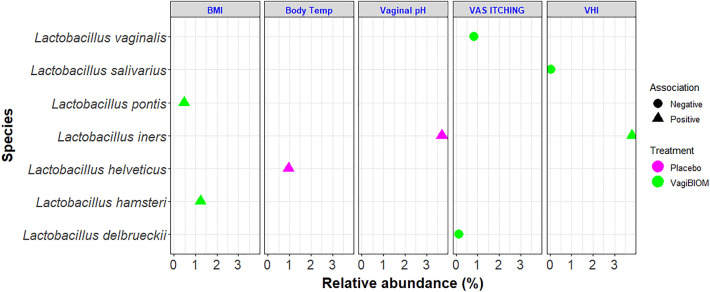
A Regression analysis between *Lactobacillus* species and efficacy parameters in the VagiBIOM- and placebo-treated groups. *Bubble points denote the relative abundance of each species; color denotes the type of treatment; triangle denotes positive association; circle denotes negative association. *Abbreviations* BMI, Body Mass Index; Body Temp, Body temperature; VAS, Visual analog scale; VHI, Vaginal health index.

A regression analysis was also performed using only *Lactobacillus* spp. in both groups after 28 days of treatment (Fig. 7). VagiBIOM post-treatment, *L. delbrueckii,* and *L. vaginalis* had a negative association with VAS itching, *L. iners* was positively associated, and *L. salivarius* was negatively associated with VHI. *Lactobacillus hamsteri* and *Limosilactobacillus pontis* were positively associated with BMI. In contrast, in the placebo-treated group, *L. helveticus* was positively associated with body temperature, and *L. iners* was found to have a positive association with vaginal pH.

### Correlation-based species network for VagiBIOM post-treatment (28th day)

**Figure 8:**
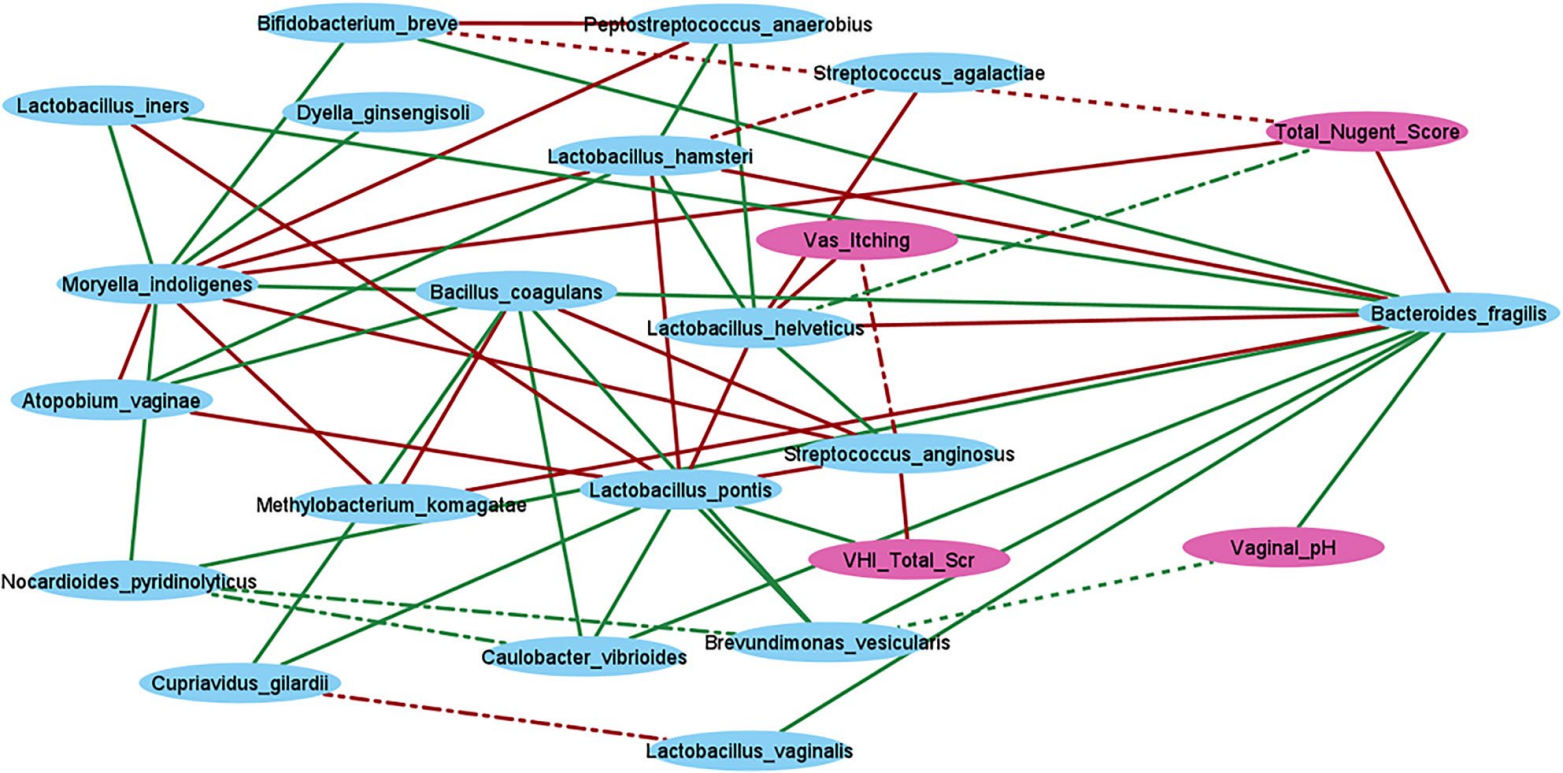
Species network post VagiBIOM treatment (Day 28). Blue nodes represent bacterial species, pink nodes represent clinical parameters, and edges represent a connection between them. Green and red lines represent positive and negative associations, respectively. All associations are significant (*p* < 0.05). *Continuous lines represent correlation coefficients ≥ 0.75 to 1 for positive associations and − 0.75 to − 1 for negative associations. Dash-dotted lines represent a correlation coefficient ≥ 0.65–0.75 for a positive association and ≥ − 0.65 to − 0.75 for a negative association. The dotted lines represent a correlation coefficient < 0.65 for a positive association < − 0.65 for a negative association. *Abbreviations* BMI, Body Mass Index; VAS, Visual analog scale; VHI, Vaginal health index.

The Spearman correlation analysis results between the top 20 most abundant species and the clinical parameters are presented in Supplementary Figures S1, S2, and S3. The correlation coefficients were used as edges, and the nodes were reflected as bacterial species or clinical parameters. A network showing only the significant associations among species and clinical parameters is shown (Fig. 8). *B. fragilis* was positively associated with vaginal pH and seven microbial species: *L. vaginalis*, *L. iners*, *B. breve*, *M. indoligenes*, *Brevundimonas vesicularis*, *Caulobacter vibrioides*, and *Nocardioides pyridinolyticus*. It was negatively associated with the total Nugent scores and three bacterial species: *L. hamsteri*, *L. helveticus*, and *M. komagatae*. Moreover, these bacterial species were positively associated with each other. *S. anginosus* was negatively associated with *Limosilactobacillus pontis* and VHI, while *Limosilactobacillus pontis* was positively associated with VHI*.*

## Discussion

We clinically evaluated the effect of the novel combination of VagiBIOM intervention on BV in perimenopausal women. BV results have demonstrated that a decline in the diversity and concentration of *Lactobacillus,* which may lead to the overgrowth of other bacterial species, predominantly BV-associated microbes. The vaginal *Lactobacillus* spp. are significant determinants of the vaginal pH, antimicrobial production, and microbial networks that thrive and compete for adhesion sites in the vaginal epithelium, thus driving localized immune responses^14,39^. Therefore, the hypothesis in the present study was that 28 days of vaginal supplementation with *Lactobacillus* spp. along with prebiotics might help modulate the beneficial microbial population, alleviate the symptoms of BV, and improve the overall vaginal health of the peri- and premenopausal female participants. The study results showed that when compared to the placebo, the VagiBIOM vaginal suppository, administered at a dose of 5 suppositories/week for 28 days, reduced the vaginal pH significantly from the baseline values. These effects were similar across other clinical parameters. However, the US Food and Drug Administration (FDA) has defined a Nugent score of < 4 as one of the requisite corroborating secondary outcomes. The responder's analysis based on this Nugent score cutoff demonstrated that the VagiBIOM post-treatment had more responders (76.08%) than the placebo (40%). The VagiBIOM post-treatment had a response as early as day 7, while the placebo post-treatment had no response on day 7, indicating the potential of VagiBIOM to modulate the vaginal microbiome rapidly.

Several previous studies have been conducted to investigate the effect of the topical administration of probiotics on the symptoms of BV^40^. We have discussed only those studies that used responder criteria similar to ours or reported a Nugent score of 0–3 to demonstrate restoration of vaginal flora after probiotic supplementation. Vaginal administration of probiotic capsules containing *Lactobacillus* spp. (*L. rhamnosus* GR-1 and *L. reuteri* RC-14 at a dose of 10^9^ per organism) for five days resulted in an average Nugent score (0–3) in 64.70% of participants, which was higher than the 33.34% in the metronidazole (0.75%) group^41^. In a similar study^27^, after seven days of intervention, 83% responded to intravaginal probiotics (> 10^9^ viable *L. brevis* CD2, *L. salivarius* FV2, and *L. plantarum* FV9), and no response was observed in the placebo. Another study reported 58.3% responders in the probiotic group and no responders in the placebo group after 28 days of intervention^42^. All the studies mentioned above were similar to ours, as they did not include a preceding phase of oral antibiotic therapy to probiotic supplementation.

A study that entailed oral clindamycin administration for seven days, followed by a vaginal *Lactobacillus* capsule containing *L. casei rhamnosus* (Lcr35, 10^9^ CFU) for seven days, reported a significant difference, with 83.1% responders compared to 35.2% in the clindamycin-treated group^43^. In another randomized controlled trial (RCT), sixty-four Brazilian women with BV received a single dose of tinidazole (2 g) supplemented with either two placebo capsules or two capsules containing *L. reuteri* RC-14 and *L. rhamnosus* GR-1 every morning for 4 weeks^44^. At the end of the study, more women exhibited "normal" vaginal microbiota (Nugent score ≤ 3) in the probiotic group than in the placebo group (75.0% vs. 34.4%; *p* = 0.011). These studies showed that the response rate was better in the studies where the participants were administered intravaginal probiotic formulation after conventional antibiotic therapy. The use of probiotics for as long as 6 months continuously or intermittently demonstrated stabilization of the vaginal ecosystem and reduction in the recurrence of BV^45–48^. Moreover, intravaginal probiotic supplementation was found to be safe during the entire study^46^.

Our study revealed a statistically significant within-group effect in the placebo group. The reason for this might be the antibacterial properties of the placebo ingredients, i.e., coconut oil fatty acids. The medium-chain fatty acids commonly found in tropical oils, such as coconut oil, have bactericidal, fungicidal, anti-protozoa, and anti-virus activities^49,50^. Coconut oil consists of 99% triglycerides with free fatty acids (less than 0.2%); in addition to lauric acid, caprylic, and capric acids, these components have been reported to exhibit antimicrobial activity^51,52^. A proposed mechanism for the effects of coconut oil suggests that fatty acids present in coconut oil can disrupt the bacterial cell membrane, leading to subsequent lysis of the bacteria^40^. Additionally, the sample size was smaller in the placebo group than in the active group, which might be one of the reasons for the high effect in this group.

Moreover, indicator species analysis revealed different bacterial species at baseline in the VagiBIOM and placebo groups. In the placebo group, *S. geniculata* and *P. alcaligenes* emerged as indicator species at baseline but disappeared after placebo treatment. *Pseudomonas alcaligenes* is an aerobic, gram-negative, mesophilic bacterium typically isolated from swimming pool water. It is used for bioremediation for oil pollution, pesticide substances, and certain chemical substances, as it can degrade polycyclic aromatic hydrocarbons. It can be an unusual human pathogen, but occurrences are infrequent. Likewise, in the VagiBIOM group, at baseline, *Actinomadura nitritigenes, M. bigeumensis, Sporobacter termitidis*, *B. saxobsidens, B. firmu*s, *A. oxalaticus*, *C. sordellii*, and *C. minus* emerged as indicator species and they are all nonpathogenic, mesophilic, and gram-positive bacteria described in the environment. For example, *B. firmu*s was significantly reduced after VagiBIOM treatment, and *B. firmus* is a nonpathogenic gram-positive bacterium of the environment with various immunomodulatory properties, particularly stimulating basal cytolytic NK cell activity against tumor cells.

On the other hand, none of these microbes were observed after 28 days of VagiBIOM treatment; *L. helveticus* and *L. hamsteri* were the two *Lactobacillus* species that emerged as indicator species. *L. helveticus* is an anaerobic, mesophilic bacterium isolated from Emmental cheese^53^. *L. helveticus* has been found to counteract *E. coli*-induced inhibition of STAT-1 tyrosine phosphorylation in three epithelial cell lines (407, Caco-2, and Hep cells)^54^. It interferes with pathogen adhesion to the uro-vaginal surfaces. *L. helveticus* KS300, a hydrogen peroxide-producing strain isolated from the human vagina, upon coculturing, reduced the viability of vaginosis-associated bacteria *Gardenella vaginalis*, *Prevotella bivia*, and uropathogenic *E. coli*^55^. *Lactobacillus hamsteri* is an anaerobic, mesophilic bacterium isolated from hamster feces^56^. In the present study, the regression analysis revealed that *L. hamsteri* had a significant negative association with vaginal pH. Both of these species effectively prevent BV by promoting the displacement of BVAM through network microbes. The species network analysis revealed that some *Lactobacillus* are crucial in determining vaginal health, as they are positively associated with the VHI and Nugent scores. The dominance of *Lactobacillus* species and the resulting health outcomes shape microbial communities in the vagina.

Interestingly, the network analysis showed that after VagiBIOM treatment, *B. fragilis* was positively associated with vaginal pH and seven bacterial species: *L. vaginalis, L. iners, B. breve, M. indoligenes, B. vesicularis, C. vibrioides,* and *N. pyridinolyticus* (Fig. 8)*.* In contrast*,* it was negatively associated with the total Nugent scores and three bacterial species: *L. hamsteri, L. helveticus*, and *M. komagatae* (Supplementary Figure S2).

*B. fragilis* has essential iron requirements regarding heme and free iron forms to support its growth in extraintestinal infections. A previous study reported that iron concentration negatively affects *Lactobacillus*^57^. A decreased pH makes iron more bioavailable, which might increase the abundance of *B. fragilis*; when their numbers increase, vaginal pH might increase, consistent with its positive association with vaginal pH. With an increase in the *B. fragilis* populations, iron will be sequestered for survival, mutually benefiting *Lactobacillus,* and this could be evidence of a transitional microbial snapshot.

After VagiBIOM treatment, *L. iners* was negatively associated with total Nugent scores and positively associated with VHI scores. In the network analysis, these variables were associated with three microbes: *M. indoligenes* (positive)*, B. fragilis* (positive), and *Limosilactobacillus pontis* (negative).

On the other hand, they were positively associated with vaginal pH and vaginal health index in the placebo-treated group. Increased *L. iners* abundance was associated with increased VHI in both study groups, making it an imperative component of the vaginal commensal flora in BV. *L. iners* was also positively associated with vaginal pH, indicating its ability to thrive well in a high pH or dysbiotic environment. This positions *L. iners* to play an imperative transitional role in restoring a healthy vaginal microenvironment in BV.

The present clinical study identified VagiBIOM as an efficacious and safe treatment option for BV-affected peri- and premenopausal women.

The limitation of this study was the smaller sample size. Mainly, the samples analyzed for microbiome studies were limited. Future studies should be aimed at a more significant multicentric cohort with defined variables such as geographical location and socioeconomic factors, BV status (with or without), and follow-up data to develop robust predictive tools that prophylactically identify target populations requiring VagiBIOM.

## Conclusion

Recurring vaginal infections are a significant quality-of-life issue for millions of women. Frequent antibiotic use may lead to antibiotic resistance, undesirable side effects, and high recurrence rates. Balancing beneficial lactobacillus in the vaginal environment may help to reduce recurrence rates; therefore, there is a growing interest in microbiome-based interventions as an alternative and adjunction therapy for vaginal infections. Our studies have found that VagiBIOM lactobacillus suppository treatment for peri- and premenopausal women with BV significantly relieved vaginal itching by decreasing vaginal pH and Nugent scores and improving the overall VHI after 4 weeks’ treatment. Our study shows, it is safe for aging women to be administered the VagiBIOM suppository intravaginally for 28 days (5 doses/week). These unique beneficial effects arise from improved *Lactobacillus* diversity. Therefore, VagiBIOM can be used as a natural microbiome-based preventative tool for mild vaginal infections and also to reseed *Lactobacillus* following antibiotic treatment for vaginal infections. Overall, the utility of VagiBIOM could be personalized across ages by evaluating its ability to reset commensal vaginal beneficial microbiome diversity.

### Supplementary Information

Supplementary Figures.

Supplementary Information.

## Data Availability

All sequencing data are publicly available in the Sequence Read Archive (SRA) under the study accession number PRJNA928846.
