# Supplementary material for: VagiBIOM Lactobacillus suppository improves vaginal health index in perimenopausal women with bacterial vaginosis: a randomized control trial
Source: Sci Rep. 2024 Feb 9;14:3317. doi: 10.1038/s41598-024-53770-1 (PMC10858244; doi:10.1038/s41598-024-53770-1)

**Supplementary Figure legends**

**Supplementary Figure 1:** S1: Correlation analysis in between Top 20 species and Clinical parameters at baseline.

**Supplementary Figure 2:** S2: Correlation analysis in between Top 20 species and Clinical parameters in VagiBIOM post-treatment.

**Supplementary Figure 3:** S3: Correlation analysis in between Top 20 species and Clinical parameters in Placebo post-treatment.

**Figure S1:**


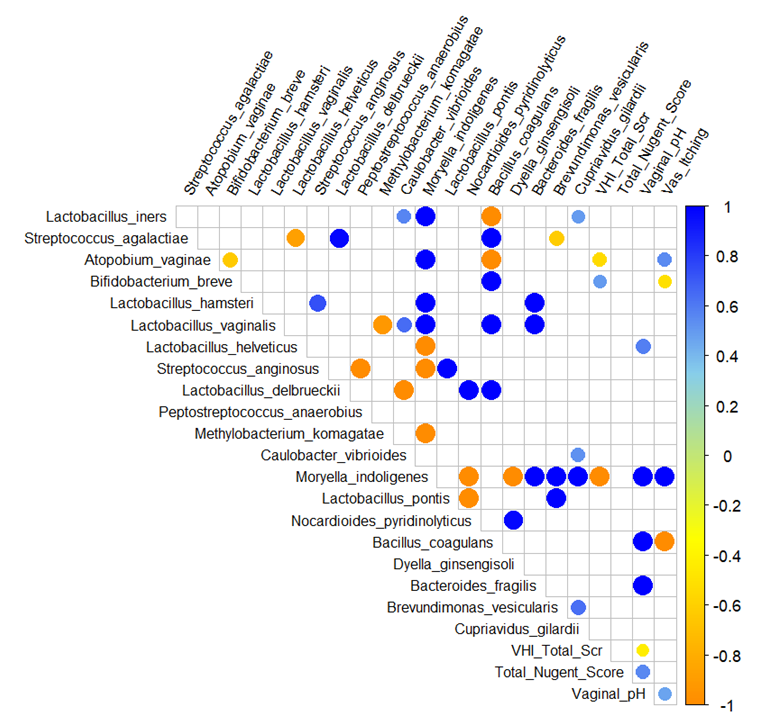


**Figure S2:**


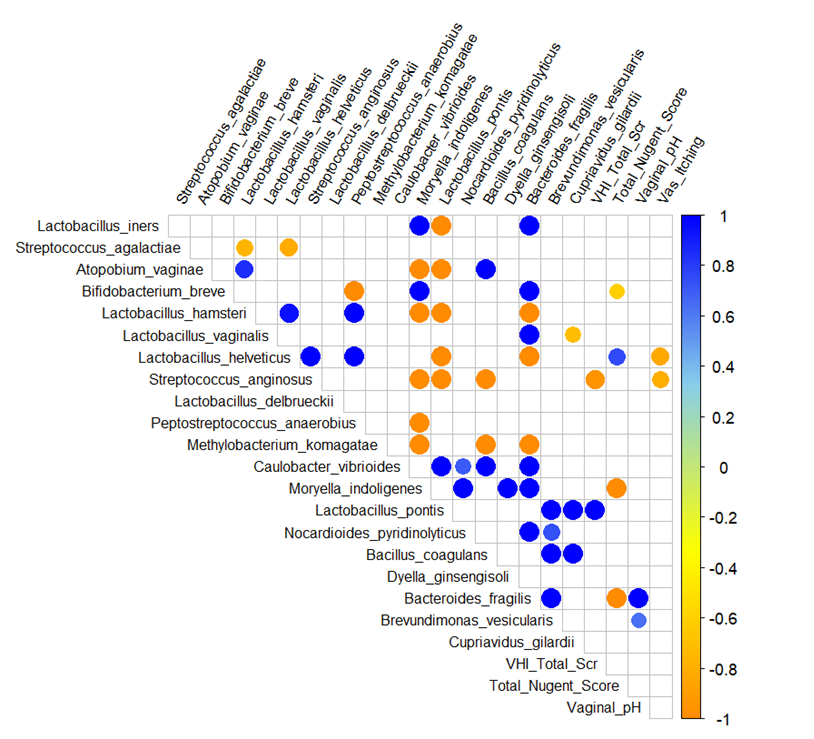


**Figure S3:**


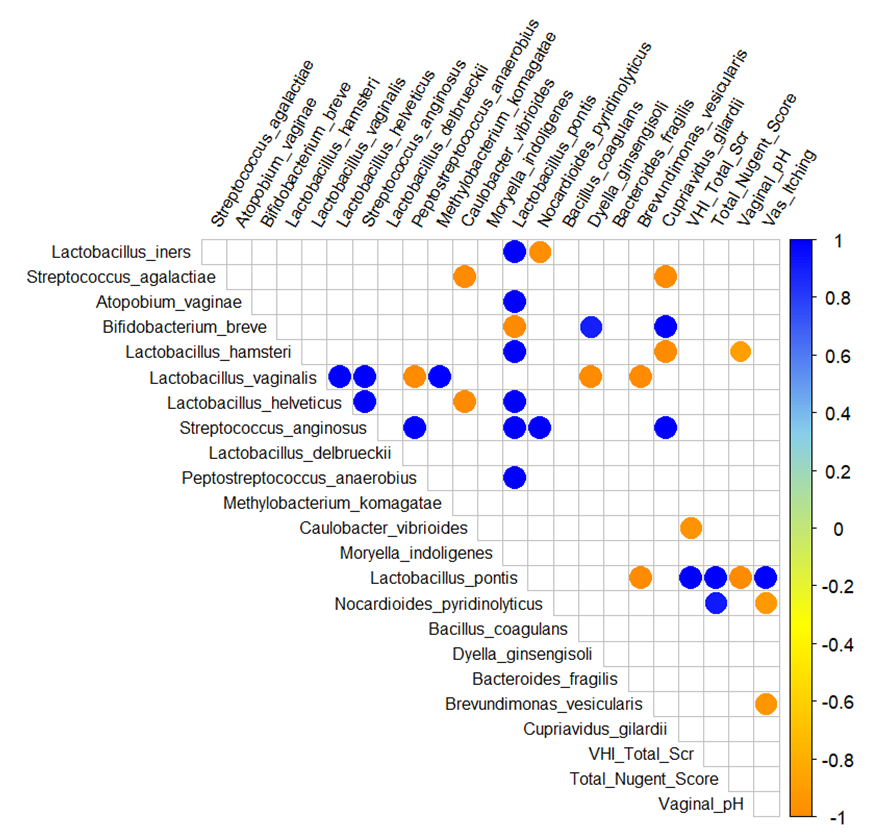

Supplement: Supplementary file 1 — Supplementary Figures. [file 41598_2024_53770_MOESM1_ESM.docx]
