# Supplementary material for: VagiBIOM Lactobacillus suppository improves vaginal health index in perimenopausal women with bacterial vaginosis: a randomized control trial
Source: Sci Rep. 2024 Feb 9;14:3317. doi: 10.1038/s41598-024-53770-1 (PMC10858244; doi:10.1038/s41598-024-53770-1)
Supplement: Supplementary file 2 — Supplementary Information. [file 41598_2024_53770_MOESM2_ESM.docx]

**Supplementary File S1**

**Inclusion criteria**

Research participants meeting **all** of the below criteria were included in the study:

- Non-pregnant, non-breastfeeding females between the ages of 40 and 65 years, inclusive.
- Participants who had at least 3 out of the following symptoms or signs:
  - Homogeneous, thin, white discharge that smoothly coats the vaginal walls;
  - Presence of the clue cells on microscopic examination
  - The pH of vaginal fluid ≥5
  - A fishy odor of vaginal discharge.
- Participants with a Nugent score of ≥ 7.
- Participants with a total Vaginal Health Index (VHI) score of <15.
- Participants with pH ≥ 5
- Willing to abstain from sexual intercourse 48 hours before the scheduled clinic visit.
- Participants who were able to comply with and perform the procedures requested by the protocol (including IP compliance, blood sample collection procedures, and study visit schedule).
- Participants who were literate enough to understand the essence of the study were informed about the purpose of the study and understood their rights.
- Participants who were able to give written informed consent and willingness to participate in the study and comply with its procedures.

**Exclusion criteria**

Participants meeting **any** of the following criteria were excluded from the trial:

- Participants with signs or symptoms of vaginal/cervical/pelvic/ urinary infection on screening or clinical diagnosis of vaginal/cervical/pelvic/ urinary infection in the past 14 days (including but not limited to yeast vulvovaginitis, chlamydia, gonorrhea, trichomonas, genital ulcer disease, pelvic inflammatory disease).
- Participants who are undergoing hormone replacement therapy.
- Participants are on prebiotics or probiotics one month prior to screening.
- Participants who were using antibiotics.
- Participants with a history/ signs of cervical or vaginal high-grade squamous intraepithelial dysplasia, atypical glandular cells of uncertain significance, or cervical intraepithelial neoplasia.
- Participants who have undergone total hysterectomy or any other surgery involving the female reproductive system.
- Participants who have been diagnosed with polycystic ovary syndrome.
- Uncontrolled type II diabetes mellitus (assessed by RBS ≥140 mg/dL.)
- Use of an immunosuppressive or immunomodulatory drug within six months prior to enrolment.
- Participants with uncontrolled hypertension defined as SBP ≥ 140 mm Hg and/or DBP ≥ 90 mm Hg.
- Abnormal TSH value from the reference range of 0.35 to 5.00 µIU/mL.
- History of any significant neurological and psychiatric condition which may affect the participation and inference of the study's endpoints.
- Participation in other clinical trials in the last three months prior to screening.
- Smokers (Past smokers can be allowed if they have abstinence for a minimum of 2 years).
- Chronic or sporadic abdominal pain, including moderate to severe dysmenorrhoea.
- Substance abuse problems (within two years) are defined as:
  - Use of recreational drugs (such as cocaine, methamphetamine, marijuana, etc.)/Nicotine dependence.
  - High-risk drinking is defined as the consumption of 4 or more alcohol-containing beverages on any day or eight or more alcohol-containing beverages per week.
- Any clinically significant illness, i.e., cardiovascular, endocrine system, immune system, respiratory system, hepatobiliary system, kidney and urinary system, neuropsychiatric, musculoskeletal, inflammatory, blood and tumors, gastrointestinal diseases, etc.
- History of hepatitis B/ hepatitis C/ HIV infection
- Regular medical treatment, including over-the-counter medications, might impact the study aims (e.g., probiotics, antibiotic drugs, laxatives, etc.)
  - Any condition that could, in the investigator's opinion, preclude the participant's ability to successfully and safely complete the study or may confound study outcomes.

**Supplementary Table**

**Table S1:** Significant changes in Vaginal pH in post-treatment groups.

| **Time point** | **pH**  **VagiBiom (N=46) Mean+S.D.** | **pH**  **Placebo (N=20)**  **(Mean+S.D.)** | **p-value** |
| --- | --- | --- | --- |
| D0 | 5.9土0.69 | 6.02土0.75 | 0.65 |
| D7 | 5.7土0.61 | 5.7土0.52 | 0.90 |
| D21 | 5.1土0.45 | 5.3土0.48 | 0.08 |
| D28 | 4.4土0.4 | 4.9土0.55 | 0.002 |

**Table S2:** Significant changes in the Nugent score in post-treatment groups.

| **Time point** | **Nugent Score**  **VagiBiom**  **(N=46) (Mean+S.D.)** | **Nugent Score**  **Placebo (N=20)**  **(Mean+S.D.)** | **p-value** |
| --- | --- | --- | --- |
| D0 | 7.8土0.76 | 7.9土0.71 | 0.71 |
| D7 | 6土1.2 | 6.3土1.03 | 0.37 |
| D21 | 4.24土1.36 | 4.65土1.04 | 0.23 |
| D28 | 2.59土1.22 | 3.65土 1.46 | 0.003 |

**Table S3:** Significant changes in VAS Itching score in post-treatment groups.

| **Time point** | **VAS Itching**  **VagiBiom (N=46) (Mean+S.D.)** | **VAS Itching**  **Placebo (N=20)**  **(Mean+S.D.)** | **p-value** |
| --- | --- | --- | --- |
| D0 | 6.4土2.7 | 6.55土2.96 | 0.87 |
| D7 | 5.28土2.27 | 5.6土2.64 | 0.62 |
| D21 | 4.17土2.22 | 4.45土2.5 | 0.65 |
| D28 | 2.78土1.81 | 3.05土1.93 | 0.59 |

**Table S4:** Significant changes in VHI score in post-treatment groups.

| **Time point** | **VHI**  **VagiBiom (N=46) (Mean+S.D.)** | **VHI**  **Placebo (N=20) (Mean+S.D.)** | **p-value** |
| --- | --- | --- | --- |
| D0 | 11.5土1.47 | 11.6土1.5 | 0.80 |
| D28 | 18.7土2.25 | 18.9土1.74 | 0.72 |
